# 1,4-Bis­(4-meth­oxy­phen­yl)naphthalene

**DOI:** 10.1107/S2414314620002126

**Published:** 2020-02-21

**Authors:** R. Manickam, G. Jagadeesan, J. Karunakaran, G. Srinivasan

**Affiliations:** a PG and Research Department of Physics, Government Arts College for Men (Autonomous), Nandanam, Chennai - 600 035, India; b Department of Physics, Jeppiar Engineering College, Jeppiar Nagar, OMR, Chennai - 600 119, India; c Department of Organic Chemistry, University of Madras, Guindy Campus, Chennai - 600 025, India; Sunway University, Malaysia

**Keywords:** crystal structure, naphthalene derivative, C—H⋯π inter­actions

## Abstract

The title naphthalene derivative, C_24_H_20_O_2_, features 4-methy­oxy-substituted benzene rings in the 1 and 4 positions of the naphthalene ring system. There are two crystallographically independent mol­ecules (*A* and *B*) in asymmetric unit. The independent mol­ecules have very similar conformations in which the naphthalene ring systems are only slightly bent, exhibiting dihedral angles between the constituent benzene rings of 3.76 (15) and 3.39 (15)° for *A* and *B*, respectively. The pendent 4-methyoxybenzene rings are splayed out of the plane through the naphthalene ring system to which they are connected [range of dihedral angles = 59.63 (13) to 67.09 (13)°]. In the crystal, the mol­ecular packing is consolidated by inter­molecular C—H⋯π inter­actions, leading to supra­molecular chains along the *b* axis. The chains assemble without directional inter­actions between them.

## Abstract 2

Two independent mol­ecules comprise the asymmetric unit of the title naphthalene derivative, which exhibit very similar conformations. The pendant 4-methyoxybenzene rings are splayed out of the plane through the naphthalene ring system.

## Graphical abstract

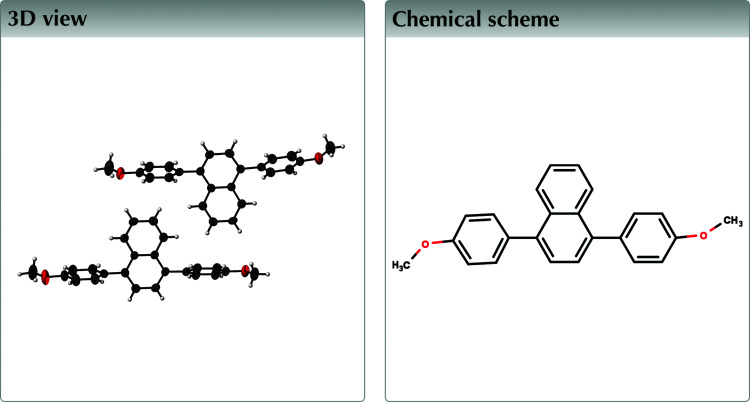

## Structure description

Mol­ecules related to the title compound are of inter­est in the field of organic electronics. A closely related structure is available whereby a perfluorinated phenyl ring is fused to the naphthalene ring system which is also perfluorinated (Tannaci *et al.*, 2008 ▸). Here, the effects of fluorination are apparent in that the pendant 4-meth­oxy­benzene rings are effectively perpendicular to the central plane.

**Figure 1:**
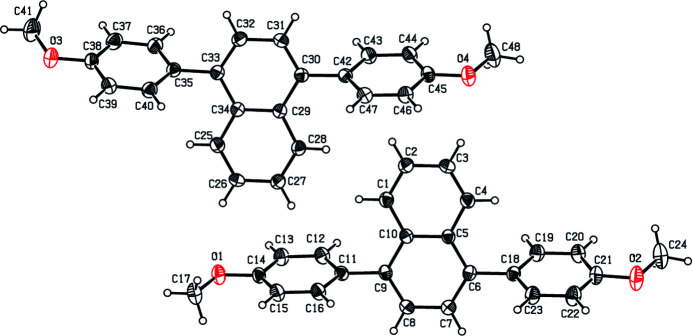
The mol­ecular structures of the title compound showing atom-numbering scheme and displacement ellipsoids at the 30% probability level. The H atoms are shown as spheres of arbitrary radius.

**Figure 2:**
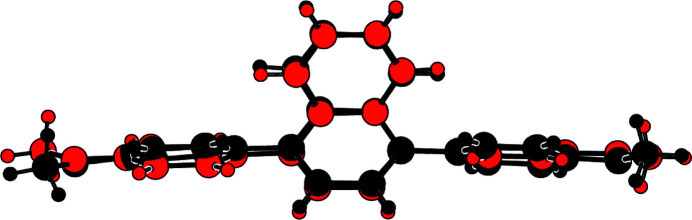
An overlay diagram of the first (red image) and inverted-second (black) independent mol­ecules of the title compound.

The mol­ecular structures of the two crystallographically independent mol­ecules comprising the asymmetric unit in the title compound are shown in Fig. 1 ▸. The mol­ecules exhibit very similar conformations, as illustrated in the overlay diagram of Fig. 2 ▸. The r.m.s deviation between the bond lengths in the two mol­ecules is 0.419 Å (Spek, 2020 ▸).

Within the naphthalene ring system, the dihedral angles between the least-squares planes through the constituent rings are 3.76 (15) and 3.39 (15)° for the two independent mol­ecules. The best plane of the (C1–C10) naphthalene ring system forms dihedral angles of 67.09 (13) and 60.71 (13)°, respectively, with the appended (C11–C16) and (C18–C23) rings of the meth­oxy-substituted benzene rings indicating splayed dispositions. The corresponding values for the second independent mol­ecule are 59.63 (13) and 63.75 (13)°. The dihedral angle between the peripheral rings, *i.e*. between the (C11–C16)/(C18–C23) benzene rings is 6.91 (16)° while that for the corresponding rings in the second independent mol­ecule, *i.e*. (C35–C40)/(C42–C47), is 8.82 (16)°.

In the crystal, C—H⋯π inter­actions, Table 1 ▸, link mol­ecules into a supra­molecular chain along the *b*-axis direction, *i.e*. with a helical topology. The chains assemble in the crystal without directional inter­actions between them.

## Synthesis and crystallization

Tetra­thia­fulvalene [2-(1,3-di­thio­lan-2-yl­idene)-2*H*-1,3-di­thi­ole; 0.204 g, 1.0 mmol] was added to a solution of 1,3-bis­(4-meth­oxy­phen­yl)isobenzo­furan (0.33 g, 1.0 mmol) in dry xylenes (15 ml). The solution was refluxed until the benzo[*c*]furan was consumed, *i.e.* after *ca* 6 h, as indicated by the disappearance of fluorescence from the solution. After removal of xylenes *in vacuo*, the crude product was dissolved in dry di­chloro­methane (DCM, 15 ml) and kept at 273 K. To this solution, triflic acid (0.075 g, 0.50 mmol) was added followed by stirring at room temperature for 10 min. After the completion of reaction (as monitored by TLC), the solution was poured into ice–water (20 ml) and then extracted with DCM (2 × 10 ml). The combined organic layer was washed with aq. NaHCO_3_ (2 × 10 ml) and then dried over Na_2_SO_4_. The removal of solvent was followed by column chromatographic purification (silica gel, 10% ethyl acetate in hexa­ne) to afford 1,4-bis­(4-meth­oxy­phen­yl)naphthalene (0.288 g, 85%) as a yellow solid. Single crystals suitable for X-ray diffraction were prepared by slow evaporation of an ethyl acetate solution of the compound held at room temperature; m.p. 421–423 K.

## Refinement

Crystal data, data collection and structure refinement details are summarized in Table 2 ▸.

## Supplementary Material

Crystal structure: contains datablock(s) I, global. DOI: 10.1107/S2414314620002126/tk4061sup1.cif

Structure factors: contains datablock(s) I. DOI: 10.1107/S2414314620002126/tk4061Isup2.hkl

Supporting information file. DOI: 10.1107/S2414314620002126/tk4061Isup3.cml

CCDC reference: 1984009

Additional supporting information:  crystallographic information; 3D view; checkCIF report

## Figures and Tables

**Table 1:** Hydrogen-bond geometry (Å, °) *Cg*1–*Cg*3 are the centroids of the (C42–C47), (C25—C34) and (C11–C16) rings, respectively.

*D*—H⋯*A*	*D*—H	H⋯*A*	*D*⋯*A*	*D*—H⋯*A*
C2—H2⋯*Cg*1	0.93	2.76	3.531 (4)	141
C15—H15⋯*Cg*2^i^	0.93	2.96	3.737 (4)	142
C27—H27⋯*Cg*3	0.93	2.81	3.610 (4)	145

Symmetry code: (i) 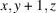 .

**Table 2:** Experimental details

Crystal data	
Chemical formula	C_24_H_20_O_2_
*M*_r_	340.40
Crystal system, space group	Monoclinic, *P*2_1_/*n*
Temperature (K)	296
*a*, *b*, *c* (Å)	21.5500 (8), 6.0366 (2), 27.4915 (9)
β (°)	92.111 (1)
*V* (Å^3^)	3573.9 (2)
*Z*	8
Radiation type	Mo *K*α
μ (mm^−1^)	0.08
Crystal size (mm)	0.20 × 0.20 × 0.15

Data collection	
Diffractometer	Bruker Kappa APEXII CCD
Absorption correction	Multi-scan (*SADABS*; Bruker, 2008 ▸)
*T*_min_, *T*_max_	0.984, 0.988
No. of measured, independent and observed [*I* > 2σ(*I*)] reflections	124530, 7894, 4154
*R*_int_	0.078
(sin θ/λ)_max_ (Å^−1^)	0.641

Refinement	
*R*[*F* ^2^ > 2σ(*F* ^2^)], *wR*(*F* ^2^), *S*	0.077, 0.281, 1.02
No. of reflections	7894
No. of parameters	473
H-atom treatment	H-atom parameters constrained
Δρ_max_, Δρ_min_ (e Å^−3^)	0.26, −0.28

Computer programs: *APEX2* and *SAINT* (Bruker, 2008 ▸), *SHELXS97* and *SHELXL97* (Sheldrick, 2008 ▸), *ORTEP-3 for Windows* (Farrugia, 2012 ▸), *Mercury* (Macrae *et al.*, 2020 ▸) and *PLATON* (Spek, 2020 ▸).

## full crystallographic data

### Crystal data

**Table:**

C_24_H_20_O_2_	*F*(000) = 1440
*M**_r_* = 340.40	*D*_x_ = 1.265 Mg m^−^^3^
Monoclinic, *P*2_1_/*n*	Mo *K*α radiation, λ = 0.71073 Å
Hall symbol: -p 2yn	Cell parameters from 7894 reflections
*a* = 21.5500 (8) Å	θ = 1.2–27.1°
*b* = 6.0366 (2) Å	µ = 0.08 mm^−^^1^
*c* = 27.4915 (9) Å	*T* = 296 K
β = 92.111 (1)°	BLOCK, yellow
*V* = 3573.9 (2) Å^3^	0.20 × 0.20 × 0.15 mm
*Z* = 8	

### Data collection

**Table:**

Bruker Kappa APEXII CCD diffractometer	7894 independent reflections
Radiation source: fine-focus sealed tube	4154 reflections with *I* > 2σ(*I*)
Graphite monochromator	*R*_int_ = 0.078
φ & ω scans	θ_max_ = 27.1°, θ_min_ = 1.2°
Absorption correction: multi-scan (SADABS; Bruker, 2008)	*h* = −27→27
*T*_min_ = 0.984, *T*_max_ = 0.988	*k* = −7→7
124530 measured reflections	*l* = −35→35

### Refinement

**Table:**

Refinement on *F*^2^	Primary atom site location: structure-invariant direct methods
Least-squares matrix: full	Secondary atom site location: difference Fourier map
*R*[*F*^2^ > 2σ(*F*^2^)] = 0.077	Hydrogen site location: inferred from neighbouring sites
*wR*(*F*^2^) = 0.281	H-atom parameters constrained
*S* = 1.02	*w* = 1/[σ^2^(*F*_o_^2^) + (0.1238*P*)^2^ + 3.8143*P*] where *P* = (*F*_o_^2^ + 2*F*_c_^2^)/3
7894 reflections	(Δ/σ)_max_ < 0.001
473 parameters	Δρ_max_ = 0.26 e Å^−^^3^
0 restraints	Δρ_min_ = −0.28 e Å^−^^3^

### Special details

**Table:**

Geometry. All esds (except the esd in the dihedral angle between two l.s. planes) are estimated using the full covariance matrix. The cell esds are taken into account individually in the estimation of esds in distances, angles and torsion angles; correlations between esds in cell parameters are only used when they are defined by crystal symmetry. An approximate (isotropic) treatment of cell esds is used for estimating esds involving l.s. planes.
Refinement. Refinement of F^2^ against ALL reflections. The weighted R-factor wR and goodness of fit S are based on F^2^, conventional R-factors R are based on F, with F set to zero for negative F^2^. The threshold expression of F^2^ > 2sigma(F^2^) is used only for calculating R-factors(gt) etc. and is not relevant to the choice of reflections for refinement. R-factors based on F^2^ are statistically about twice as large as those based on F, and R- factors based on ALL data will be even larger. The C-bound H-atoms were included in calculated positions and treated as riding with C—H = 0.93–0.96 Å, and with *U*_iso_(H) = 1.5*U*_eq_(*C*-methyl) and 1.2*U*_eq_(C) for the other H-atoms.

### Fractional atomic coordinates and isotropic or equivalent isotropic displacement parameters (Å^2^)

**Table:**

	*x*	*y*	*z*	*U*_iso_*/*U*_eq_	
C1	0.42322 (15)	0.6450 (5)	0.76704 (11)	0.0430 (8)	
H1	0.4453	0.6431	0.7386	0.052*	
C2	0.43579 (16)	0.4887 (6)	0.80184 (11)	0.0468 (8)	
H2	0.4656	0.3804	0.7969	0.056*	
C3	0.40344 (16)	0.4927 (6)	0.84511 (12)	0.0485 (8)	
H3	0.4122	0.3874	0.8691	0.058*	
C4	0.35946 (15)	0.6486 (5)	0.85236 (11)	0.0442 (8)	
H4	0.3389	0.6492	0.8815	0.053*	
C5	0.34414 (14)	0.8104 (5)	0.81670 (10)	0.0357 (7)	
C6	0.29864 (14)	0.9775 (5)	0.82446 (10)	0.0379 (7)	
C7	0.29017 (16)	1.1416 (5)	0.78998 (11)	0.0457 (8)	
H7	0.2614	1.2534	0.7950	0.055*	
C8	0.32445 (15)	1.1425 (5)	0.74734 (11)	0.0443 (8)	
H8	0.3182	1.2574	0.7252	0.053*	
C9	0.36667 (14)	0.9803 (5)	0.73731 (10)	0.0378 (7)	
C10	0.37770 (14)	0.8092 (5)	0.77290 (10)	0.0360 (7)	
C11	0.40148 (14)	0.9863 (5)	0.69146 (10)	0.0392 (7)	
C12	0.39530 (16)	0.8223 (6)	0.65583 (11)	0.0456 (8)	
H12	0.3686	0.7039	0.6605	0.055*	
C13	0.42824 (16)	0.8328 (6)	0.61361 (11)	0.0469 (8)	
H13	0.4231	0.7231	0.5901	0.056*	
C14	0.46885 (15)	1.0066 (6)	0.60642 (10)	0.0419 (8)	
C15	0.47614 (17)	1.1689 (6)	0.64097 (11)	0.0495 (9)	
H15	0.5035	1.2856	0.6364	0.059*	
C16	0.44226 (17)	1.1576 (5)	0.68292 (11)	0.0478 (8)	
H16	0.4472	1.2692	0.7061	0.057*	
C17	0.5414 (2)	1.1779 (7)	0.55437 (13)	0.0680 (11)	
H17A	0.5197	1.3165	0.5561	0.102*	
H17B	0.5577	1.1606	0.5226	0.102*	
H17C	0.5749	1.1758	0.5784	0.102*	
C18	0.26124 (14)	0.9741 (5)	0.86889 (10)	0.0386 (7)	
C19	0.22387 (15)	0.7959 (6)	0.87984 (11)	0.0463 (8)	
H19	0.2223	0.6748	0.8589	0.056*	
C20	0.18858 (16)	0.7924 (6)	0.92110 (11)	0.0494 (9)	
H20	0.1637	0.6709	0.9276	0.059*	
C21	0.19088 (16)	0.9707 (6)	0.95229 (11)	0.0506 (9)	
C22	0.22778 (18)	1.1493 (6)	0.94256 (13)	0.0584 (10)	
H22	0.2296	1.2691	0.9638	0.070*	
C23	0.26234 (17)	1.1513 (6)	0.90108 (12)	0.0526 (9)	
H23	0.2868	1.2740	0.8946	0.063*	
C24	0.1212 (2)	0.8005 (9)	1.00620 (15)	0.0839 (15)	
H24A	0.0918	0.7689	0.9800	0.126*	
H24B	0.0994	0.8323	1.0352	0.126*	
H24C	0.1477	0.6745	1.0117	0.126*	
C25	0.64533 (16)	0.5039 (5)	0.64015 (11)	0.0449 (8)	
H25	0.6665	0.4996	0.6113	0.054*	
C26	0.60133 (16)	0.6621 (5)	0.64606 (11)	0.0462 (8)	
H26	0.5934	0.7659	0.6216	0.055*	
C27	0.56793 (16)	0.6694 (6)	0.68881 (11)	0.0461 (8)	
H27	0.5381	0.7785	0.6928	0.055*	
C28	0.57920 (15)	0.5166 (5)	0.72438 (11)	0.0424 (8)	
H28	0.5562	0.5220	0.7523	0.051*	
C29	0.62471 (14)	0.3499 (5)	0.72018 (10)	0.0362 (7)	
C30	0.63511 (14)	0.1831 (5)	0.75665 (10)	0.0371 (7)	
C31	0.67742 (16)	0.0207 (5)	0.74832 (11)	0.0452 (8)	
H31	0.6829	−0.0925	0.7710	0.054*	
C32	0.71299 (16)	0.0197 (6)	0.70637 (11)	0.0462 (8)	
H32	0.7421	−0.0921	0.7026	0.055*	
C33	0.70595 (14)	0.1781 (5)	0.67099 (10)	0.0378 (7)	
C34	0.65981 (14)	0.3453 (5)	0.67696 (10)	0.0366 (7)	
C35	0.74535 (15)	0.1737 (5)	0.62787 (11)	0.0403 (7)	
C36	0.74759 (17)	−0.0110 (6)	0.59848 (12)	0.0491 (8)	
H36	0.7223	−0.1314	0.6051	0.059*	
C37	0.78635 (18)	−0.0229 (6)	0.55932 (13)	0.0561 (9)	
H37	0.7863	−0.1480	0.5396	0.067*	
C38	0.82476 (17)	0.1528 (6)	0.55015 (12)	0.0517 (9)	
C39	0.82314 (17)	0.3408 (6)	0.57853 (12)	0.0523 (9)	
H39	0.8483	0.4610	0.5717	0.063*	
C40	0.78406 (16)	0.3503 (6)	0.61715 (12)	0.0473 (8)	
H40	0.7836	0.4771	0.6364	0.057*	
C41	0.8757 (3)	−0.0372 (9)	0.48697 (16)	0.0996 (18)	
H41A	0.8867	−0.1565	0.5087	0.149*	
H41B	0.9083	−0.0150	0.4646	0.149*	
H41C	0.8379	−0.0733	0.4691	0.149*	
C42	0.59868 (14)	0.1807 (5)	0.80161 (10)	0.0382 (7)	
C43	0.56057 (17)	0.0038 (5)	0.81173 (12)	0.0481 (8)	
H43	0.5577	−0.1130	0.7897	0.058*	
C44	0.52641 (17)	−0.0064 (6)	0.85351 (11)	0.0492 (8)	
H44	0.5008	−0.1269	0.8591	0.059*	
C45	0.53109 (15)	0.1648 (5)	0.88663 (10)	0.0423 (8)	
C46	0.56849 (16)	0.3437 (6)	0.87743 (11)	0.0449 (8)	
H46	0.5711	0.4602	0.8995	0.054*	
C47	0.60218 (15)	0.3520 (5)	0.83579 (11)	0.0429 (8)	
H47	0.6276	0.4734	0.8304	0.051*	
C48	0.4601 (2)	−0.0070 (8)	0.93981 (16)	0.0840 (14)	
H48A	0.4298	−0.0267	0.9137	0.126*	
H48B	0.4394	0.0220	0.9695	0.126*	
H48C	0.4847	−0.1390	0.9435	0.126*	
O1	0.49974 (12)	1.0013 (4)	0.56346 (8)	0.0566 (7)	
O2	0.15737 (14)	0.9849 (5)	0.99390 (9)	0.0790 (9)	
O3	0.86713 (14)	0.1572 (5)	0.51385 (10)	0.0776 (9)	
O4	0.49915 (12)	0.1742 (4)	0.92913 (8)	0.0601 (7)	

### Atomic displacement parameters (Å^2^)

**Table:**

	*U*^11^	*U*^22^	*U*^33^	*U*^12^	*U*^13^	*U*^23^
C1	0.0435 (19)	0.0480 (19)	0.0377 (16)	0.0002 (16)	0.0024 (14)	−0.0006 (14)
C2	0.046 (2)	0.0478 (19)	0.0462 (18)	0.0056 (16)	0.0030 (15)	0.0003 (15)
C3	0.052 (2)	0.049 (2)	0.0444 (18)	0.0091 (17)	−0.0002 (15)	0.0116 (15)
C4	0.0435 (19)	0.0504 (19)	0.0387 (17)	0.0019 (16)	0.0013 (14)	0.0047 (15)
C5	0.0354 (16)	0.0401 (16)	0.0315 (15)	−0.0007 (14)	0.0003 (12)	0.0005 (12)
C6	0.0361 (17)	0.0430 (17)	0.0345 (15)	−0.0030 (14)	0.0012 (13)	−0.0061 (13)
C7	0.049 (2)	0.0444 (18)	0.0445 (18)	0.0045 (16)	0.0057 (15)	0.0011 (15)
C8	0.050 (2)	0.0441 (18)	0.0386 (17)	0.0036 (16)	0.0013 (14)	0.0074 (14)
C9	0.0399 (17)	0.0438 (17)	0.0298 (15)	−0.0032 (15)	0.0001 (13)	−0.0003 (13)
C10	0.0377 (17)	0.0385 (16)	0.0316 (15)	−0.0035 (14)	−0.0012 (12)	−0.0008 (12)
C11	0.0422 (18)	0.0429 (18)	0.0322 (15)	0.0007 (15)	0.0000 (13)	0.0025 (13)
C12	0.049 (2)	0.0512 (19)	0.0364 (16)	−0.0076 (16)	−0.0023 (14)	−0.0017 (15)
C13	0.054 (2)	0.053 (2)	0.0334 (16)	−0.0058 (17)	−0.0008 (14)	−0.0100 (15)
C14	0.0452 (19)	0.0519 (19)	0.0288 (15)	0.0021 (16)	0.0032 (13)	0.0017 (14)
C15	0.058 (2)	0.049 (2)	0.0422 (18)	−0.0116 (17)	0.0083 (16)	−0.0018 (15)
C16	0.065 (2)	0.0427 (18)	0.0357 (16)	−0.0100 (17)	0.0066 (15)	−0.0064 (14)
C17	0.074 (3)	0.081 (3)	0.050 (2)	−0.015 (2)	0.019 (2)	0.003 (2)
C18	0.0351 (17)	0.0437 (17)	0.0370 (16)	0.0006 (14)	0.0012 (13)	−0.0016 (13)
C19	0.047 (2)	0.0501 (19)	0.0420 (17)	−0.0064 (16)	0.0039 (15)	−0.0079 (15)
C20	0.047 (2)	0.059 (2)	0.0424 (18)	−0.0118 (17)	0.0060 (15)	−0.0031 (16)
C21	0.049 (2)	0.066 (2)	0.0374 (17)	−0.0028 (19)	0.0113 (15)	−0.0043 (16)
C22	0.066 (2)	0.059 (2)	0.051 (2)	−0.013 (2)	0.0132 (18)	−0.0207 (18)
C23	0.057 (2)	0.052 (2)	0.0493 (19)	−0.0100 (18)	0.0128 (17)	−0.0088 (16)
C24	0.078 (3)	0.116 (4)	0.059 (3)	−0.024 (3)	0.026 (2)	0.001 (3)
C25	0.0458 (19)	0.0479 (19)	0.0409 (17)	0.0011 (16)	0.0035 (14)	0.0043 (15)
C26	0.050 (2)	0.0440 (18)	0.0443 (18)	0.0094 (16)	−0.0009 (15)	0.0122 (15)
C27	0.046 (2)	0.0485 (19)	0.0440 (18)	0.0077 (16)	0.0039 (15)	0.0007 (15)
C28	0.0443 (19)	0.0447 (18)	0.0382 (16)	0.0018 (16)	0.0031 (14)	−0.0010 (14)
C29	0.0359 (17)	0.0356 (16)	0.0368 (16)	−0.0027 (14)	0.0007 (13)	−0.0039 (13)
C30	0.0376 (17)	0.0391 (17)	0.0343 (15)	−0.0002 (14)	−0.0017 (13)	−0.0005 (13)
C31	0.052 (2)	0.0430 (18)	0.0411 (17)	0.0063 (16)	0.0026 (15)	0.0075 (14)
C32	0.045 (2)	0.0456 (19)	0.0484 (19)	0.0078 (16)	0.0032 (15)	−0.0010 (15)
C33	0.0375 (17)	0.0402 (17)	0.0358 (15)	−0.0003 (14)	0.0003 (13)	−0.0026 (13)
C34	0.0351 (16)	0.0387 (16)	0.0357 (15)	−0.0008 (14)	−0.0008 (12)	−0.0025 (13)
C35	0.0400 (18)	0.0430 (18)	0.0378 (16)	0.0041 (15)	0.0013 (13)	0.0002 (14)
C36	0.052 (2)	0.0454 (19)	0.0501 (19)	0.0012 (17)	0.0084 (16)	−0.0033 (16)
C37	0.066 (3)	0.054 (2)	0.048 (2)	0.009 (2)	0.0048 (18)	−0.0102 (17)
C38	0.052 (2)	0.064 (2)	0.0405 (18)	0.0125 (19)	0.0116 (16)	0.0080 (17)
C39	0.052 (2)	0.053 (2)	0.052 (2)	−0.0006 (18)	0.0096 (16)	0.0072 (17)
C40	0.048 (2)	0.0461 (19)	0.0477 (19)	−0.0004 (16)	0.0043 (15)	−0.0035 (15)
C41	0.126 (5)	0.119 (4)	0.056 (3)	0.029 (4)	0.033 (3)	−0.009 (3)
C42	0.0393 (18)	0.0422 (17)	0.0327 (15)	0.0039 (15)	−0.0022 (13)	0.0007 (13)
C43	0.062 (2)	0.0400 (18)	0.0430 (18)	−0.0083 (17)	0.0067 (16)	−0.0051 (14)
C44	0.059 (2)	0.0448 (19)	0.0444 (18)	−0.0086 (17)	0.0072 (16)	0.0038 (15)
C45	0.0447 (19)	0.0513 (19)	0.0309 (15)	−0.0027 (16)	0.0003 (13)	0.0015 (14)
C46	0.050 (2)	0.0495 (19)	0.0345 (16)	−0.0049 (17)	−0.0035 (14)	−0.0091 (14)
C47	0.0418 (19)	0.0453 (18)	0.0412 (17)	−0.0054 (15)	−0.0034 (14)	−0.0024 (14)
C48	0.100 (4)	0.088 (3)	0.066 (3)	−0.027 (3)	0.033 (3)	0.004 (2)
O1	0.0658 (17)	0.0693 (16)	0.0355 (12)	−0.0087 (14)	0.0114 (11)	−0.0048 (11)
O2	0.087 (2)	0.096 (2)	0.0561 (16)	−0.0194 (18)	0.0347 (15)	−0.0180 (15)
O3	0.083 (2)	0.090 (2)	0.0618 (17)	0.0176 (17)	0.0337 (15)	0.0066 (16)
O4	0.0695 (17)	0.0706 (17)	0.0412 (13)	−0.0107 (14)	0.0150 (12)	−0.0020 (12)

### Geometric parameters (Å, º)

**Table:**

C1—C2	1.364 (4)	C25—C26	1.360 (4)
C1—C10	1.408 (4)	C25—C34	1.419 (4)
C1—H1	0.9300	C25—H25	0.9300
C2—C3	1.401 (4)	C26—C27	1.401 (4)
C2—H2	0.9300	C26—H26	0.9300
C3—C4	1.356 (4)	C27—C28	1.360 (4)
C3—H3	0.9300	C27—H27	0.9300
C4—C5	1.414 (4)	C28—C29	1.413 (4)
C4—H4	0.9300	C28—H28	0.9300
C5—C10	1.427 (4)	C29—C34	1.432 (4)
C5—C6	1.428 (4)	C29—C30	1.433 (4)
C6—C7	1.379 (4)	C30—C31	1.364 (4)
C6—C18	1.488 (4)	C30—C42	1.488 (4)
C7—C8	1.409 (4)	C31—C32	1.408 (4)
C7—H7	0.9300	C31—H31	0.9300
C8—C9	1.372 (4)	C32—C33	1.369 (4)
C8—H8	0.9300	C32—H32	0.9300
C9—C10	1.437 (4)	C33—C34	1.431 (4)
C9—C11	1.491 (4)	C33—C35	1.484 (4)
C11—C16	1.383 (4)	C35—C36	1.379 (4)
C11—C12	1.395 (4)	C35—C40	1.392 (4)
C12—C13	1.384 (4)	C36—C37	1.389 (5)
C12—H12	0.9300	C36—H36	0.9300
C13—C14	1.385 (5)	C37—C38	1.375 (5)
C13—H13	0.9300	C37—H37	0.9300
C14—C15	1.369 (4)	C38—O3	1.378 (4)
C14—O1	1.377 (3)	C38—C39	1.378 (5)
C15—C16	1.389 (4)	C39—C40	1.380 (4)
C15—H15	0.9300	C39—H39	0.9300
C16—H16	0.9300	C40—H40	0.9300
C17—O1	1.422 (4)	C41—O3	1.403 (5)
C17—H17A	0.9600	C41—H41A	0.9600
C17—H17B	0.9600	C41—H41B	0.9600
C17—H17C	0.9600	C41—H41C	0.9600
C18—C19	1.383 (4)	C42—C43	1.382 (4)
C18—C23	1.388 (4)	C42—C47	1.398 (4)
C19—C20	1.389 (4)	C43—C44	1.388 (4)
C19—H19	0.9300	C43—H43	0.9300
C20—C21	1.375 (5)	C44—C45	1.379 (4)
C20—H20	0.9300	C44—H44	0.9300
C21—C22	1.372 (5)	C45—C46	1.377 (4)
C21—O2	1.378 (4)	C45—O4	1.379 (3)
C22—C23	1.385 (5)	C46—C47	1.379 (4)
C22—H22	0.9300	C46—H46	0.9300
C23—H23	0.9300	C47—H47	0.9300
C24—O2	1.407 (5)	C48—O4	1.418 (5)
C24—H24A	0.9600	C48—H48A	0.9600
C24—H24B	0.9600	C48—H48B	0.9600
C24—H24C	0.9600	C48—H48C	0.9600
			
C2—C1—C10	121.9 (3)	C34—C25—H25	119.2
C2—C1—H1	119.1	C25—C26—C27	120.3 (3)
C10—C1—H1	119.1	C25—C26—H26	119.9
C1—C2—C3	119.4 (3)	C27—C26—H26	119.9
C1—C2—H2	120.3	C28—C27—C26	119.9 (3)
C3—C2—H2	120.3	C28—C27—H27	120.1
C4—C3—C2	120.6 (3)	C26—C27—H27	120.1
C4—C3—H3	119.7	C27—C28—C29	122.1 (3)
C2—C3—H3	119.7	C27—C28—H28	119.0
C3—C4—C5	121.6 (3)	C29—C28—H28	119.0
C3—C4—H4	119.2	C28—C29—C34	118.1 (3)
C5—C4—H4	119.2	C28—C29—C30	122.3 (3)
C4—C5—C10	117.9 (3)	C34—C29—C30	119.6 (3)
C4—C5—C6	121.9 (3)	C31—C30—C29	118.4 (3)
C10—C5—C6	120.1 (3)	C31—C30—C42	120.6 (3)
C7—C6—C5	118.6 (3)	C29—C30—C42	121.0 (3)
C7—C6—C18	120.8 (3)	C30—C31—C32	121.9 (3)
C5—C6—C18	120.6 (3)	C30—C31—H31	119.1
C6—C7—C8	121.0 (3)	C32—C31—H31	119.1
C6—C7—H7	119.5	C33—C32—C31	122.0 (3)
C8—C7—H7	119.5	C33—C32—H32	119.0
C9—C8—C7	122.5 (3)	C31—C32—H32	119.0
C9—C8—H8	118.8	C32—C33—C34	118.1 (3)
C7—C8—H8	118.8	C32—C33—C35	120.2 (3)
C8—C9—C10	118.1 (3)	C34—C33—C35	121.7 (3)
C8—C9—C11	120.7 (3)	C25—C34—C33	122.0 (3)
C10—C9—C11	121.2 (3)	C25—C34—C29	118.0 (3)
C1—C10—C5	118.5 (3)	C33—C34—C29	119.9 (3)
C1—C10—C9	121.8 (3)	C36—C35—C40	117.5 (3)
C5—C10—C9	119.6 (3)	C36—C35—C33	121.1 (3)
C16—C11—C12	117.2 (3)	C40—C35—C33	121.4 (3)
C16—C11—C9	120.3 (3)	C35—C36—C37	122.1 (3)
C12—C11—C9	122.6 (3)	C35—C36—H36	119.0
C13—C12—C11	121.2 (3)	C37—C36—H36	119.0
C13—C12—H12	119.4	C38—C37—C36	119.1 (3)
C11—C12—H12	119.4	C38—C37—H37	120.5
C12—C13—C14	120.0 (3)	C36—C37—H37	120.5
C12—C13—H13	120.0	C37—C38—O3	124.6 (3)
C14—C13—H13	120.0	C37—C38—C39	120.2 (3)
C15—C14—O1	124.4 (3)	O3—C38—C39	115.2 (3)
C15—C14—C13	120.0 (3)	C38—C39—C40	119.9 (3)
O1—C14—C13	115.6 (3)	C38—C39—H39	120.0
C14—C15—C16	119.4 (3)	C40—C39—H39	120.0
C14—C15—H15	120.3	C39—C40—C35	121.2 (3)
C16—C15—H15	120.3	C39—C40—H40	119.4
C11—C16—C15	122.3 (3)	C35—C40—H40	119.4
C11—C16—H16	118.9	O3—C41—H41A	109.5
C15—C16—H16	118.9	O3—C41—H41B	109.5
O1—C17—H17A	109.5	H41A—C41—H41B	109.5
O1—C17—H17B	109.5	O3—C41—H41C	109.5
H17A—C17—H17B	109.5	H41A—C41—H41C	109.5
O1—C17—H17C	109.5	H41B—C41—H41C	109.5
H17A—C17—H17C	109.5	C43—C42—C47	117.0 (3)
H17B—C17—H17C	109.5	C43—C42—C30	120.7 (3)
C19—C18—C23	117.2 (3)	C47—C42—C30	122.3 (3)
C19—C18—C6	121.8 (3)	C42—C43—C44	122.6 (3)
C23—C18—C6	121.0 (3)	C42—C43—H43	118.7
C18—C19—C20	122.0 (3)	C44—C43—H43	118.7
C18—C19—H19	119.0	C45—C44—C43	119.0 (3)
C20—C19—H19	119.0	C45—C44—H44	120.5
C21—C20—C19	119.3 (3)	C43—C44—H44	120.5
C21—C20—H20	120.4	C46—C45—O4	115.9 (3)
C19—C20—H20	120.4	C46—C45—C44	119.7 (3)
C22—C21—C20	120.1 (3)	O4—C45—C44	124.3 (3)
C22—C21—O2	116.0 (3)	C45—C46—C47	120.7 (3)
C20—C21—O2	124.0 (3)	C45—C46—H46	119.6
C21—C22—C23	120.0 (3)	C47—C46—H46	119.6
C21—C22—H22	120.0	C46—C47—C42	120.9 (3)
C23—C22—H22	120.0	C46—C47—H47	119.5
C22—C23—C18	121.4 (3)	C42—C47—H47	119.5
C22—C23—H23	119.3	O4—C48—H48A	109.5
C18—C23—H23	119.3	O4—C48—H48B	109.5
O2—C24—H24A	109.5	H48A—C48—H48B	109.5
O2—C24—H24B	109.5	O4—C48—H48C	109.5
H24A—C24—H24B	109.5	H48A—C48—H48C	109.5
O2—C24—H24C	109.5	H48B—C48—H48C	109.5
H24A—C24—H24C	109.5	C14—O1—C17	117.5 (3)
H24B—C24—H24C	109.5	C21—O2—C24	117.5 (3)
C26—C25—C34	121.6 (3)	C38—O3—C41	118.0 (4)
C26—C25—H25	119.2	C45—O4—C48	117.5 (3)
			
C10—C1—C2—C3	−1.1 (5)	C27—C28—C29—C30	−177.5 (3)
C1—C2—C3—C4	0.7 (5)	C28—C29—C30—C31	176.2 (3)
C2—C3—C4—C5	0.7 (5)	C34—C29—C30—C31	−1.6 (4)
C3—C4—C5—C10	−1.7 (5)	C28—C29—C30—C42	−1.2 (4)
C3—C4—C5—C6	−178.9 (3)	C34—C29—C30—C42	−179.0 (3)
C4—C5—C6—C7	174.2 (3)	C29—C30—C31—C32	3.3 (5)
C10—C5—C6—C7	−3.0 (4)	C42—C30—C31—C32	−179.4 (3)
C4—C5—C6—C18	−5.8 (4)	C30—C31—C32—C33	−1.8 (5)
C10—C5—C6—C18	177.1 (3)	C31—C32—C33—C34	−1.5 (5)
C5—C6—C7—C8	1.5 (5)	C31—C32—C33—C35	178.6 (3)
C18—C6—C7—C8	−178.5 (3)	C26—C25—C34—C33	179.9 (3)
C6—C7—C8—C9	1.4 (5)	C26—C25—C34—C29	2.6 (5)
C7—C8—C9—C10	−2.6 (5)	C32—C33—C34—C25	−174.1 (3)
C7—C8—C9—C11	179.3 (3)	C35—C33—C34—C25	5.7 (5)
C2—C1—C10—C5	0.1 (5)	C32—C33—C34—C29	3.0 (4)
C2—C1—C10—C9	176.8 (3)	C35—C33—C34—C29	−177.1 (3)
C4—C5—C10—C1	1.3 (4)	C28—C29—C34—C25	−2.1 (4)
C6—C5—C10—C1	178.5 (3)	C30—C29—C34—C25	175.8 (3)
C4—C5—C10—C9	−175.5 (3)	C28—C29—C34—C33	−179.4 (3)
C6—C5—C10—C9	1.7 (4)	C30—C29—C34—C33	−1.5 (4)
C8—C9—C10—C1	−175.6 (3)	C32—C33—C35—C36	56.0 (4)
C11—C9—C10—C1	2.4 (4)	C34—C33—C35—C36	−123.9 (3)
C8—C9—C10—C5	1.1 (4)	C32—C33—C35—C40	−120.3 (4)
C11—C9—C10—C5	179.1 (3)	C34—C33—C35—C40	59.8 (4)
C8—C9—C11—C16	63.9 (4)	C40—C35—C36—C37	−0.4 (5)
C10—C9—C11—C16	−114.1 (4)	C33—C35—C36—C37	−176.8 (3)
C8—C9—C11—C12	−116.7 (4)	C35—C36—C37—C38	1.5 (5)
C10—C9—C11—C12	65.3 (4)	C36—C37—C38—O3	177.0 (3)
C16—C11—C12—C13	−0.6 (5)	C36—C37—C38—C39	−2.2 (5)
C9—C11—C12—C13	179.9 (3)	C37—C38—C39—C40	1.8 (5)
C11—C12—C13—C14	0.9 (5)	O3—C38—C39—C40	−177.5 (3)
C12—C13—C14—C15	−0.4 (5)	C38—C39—C40—C35	−0.8 (5)
C12—C13—C14—O1	179.5 (3)	C36—C35—C40—C39	0.1 (5)
O1—C14—C15—C16	179.7 (3)	C33—C35—C40—C39	176.5 (3)
C13—C14—C15—C16	−0.3 (5)	C31—C30—C42—C43	−59.6 (4)
C12—C11—C16—C15	−0.1 (5)	C29—C30—C42—C43	117.7 (3)
C9—C11—C16—C15	179.3 (3)	C31—C30—C42—C47	119.0 (3)
C14—C15—C16—C11	0.6 (5)	C29—C30—C42—C47	−63.7 (4)
C7—C6—C18—C19	120.9 (4)	C47—C42—C43—C44	0.5 (5)
C5—C6—C18—C19	−59.1 (4)	C30—C42—C43—C44	179.2 (3)
C7—C6—C18—C23	−58.8 (4)	C42—C43—C44—C45	−0.8 (5)
C5—C6—C18—C23	121.2 (3)	C43—C44—C45—C46	1.1 (5)
C23—C18—C19—C20	0.1 (5)	C43—C44—C45—O4	179.4 (3)
C6—C18—C19—C20	−179.6 (3)	O4—C45—C46—C47	−179.6 (3)
C18—C19—C20—C21	−0.3 (5)	C44—C45—C46—C47	−1.1 (5)
C19—C20—C21—C22	−0.1 (6)	C45—C46—C47—C42	0.8 (5)
C19—C20—C21—O2	179.4 (3)	C43—C42—C47—C46	−0.5 (5)
C20—C21—C22—C23	0.6 (6)	C30—C42—C47—C46	−179.1 (3)
O2—C21—C22—C23	−178.9 (3)	C15—C14—O1—C17	−0.7 (5)
C21—C22—C23—C18	−0.8 (6)	C13—C14—O1—C17	179.4 (3)
C19—C18—C23—C22	0.4 (5)	C22—C21—O2—C24	−177.2 (4)
C6—C18—C23—C22	−179.9 (3)	C20—C21—O2—C24	3.4 (6)
C34—C25—C26—C27	−1.3 (5)	C37—C38—O3—C41	−5.7 (6)
C25—C26—C27—C28	−0.5 (5)	C39—C38—O3—C41	173.5 (4)
C26—C27—C28—C29	1.0 (5)	C46—C45—O4—C48	179.9 (3)
C27—C28—C29—C34	0.4 (5)	C44—C45—O4—C48	1.5 (5)

### Hydrogen-bond geometry (Å, º)

Cg1–Cg3 are the centroids of the (C42–C47), (C25—C34) and (C11–C16) rings, respectively.

**Table:**

*D*—H···*A*	*D*—H	H···*A*	*D*···*A*	*D*—H···*A*
C2—H2···*Cg*1	0.93	2.76	3.531 (4)	141
C15—H15···*Cg*2^i^	0.93	2.96	3.737 (4)	142
C27—H27···*Cg*3	0.93	2.81	3.610 (4)	145

Symmetry code: (i) *x*, *y*+1, *z*.

## References

[bb1] Bruker (2008). *APEX2*, *SAINT* and *SADABS*. Bruker AXS Inc., Madison, Wisconsin, USA.

[bb2] Farrugia, L. J. (2012). *J. Appl. Cryst.* **45**, 849–854.

[bb3] Macrae, C. F., Sovago, I., Cottrell, S. J., Galek, P. T. A., McCabe, P., Pidcock, E., Platings, M., Shields, G. P., Stevens, J. S., Towler, M. & Wood, P. A. (2020). *J. Appl. Cryst.* **53**, 226–235. 10.1107/S1600576719014092 PMC6998782 32047413

[bb4] Sheldrick, G. M. (2008). *Acta Cryst.* A**64**, 112–122. 10.1107/S0108767307043930 18156677

[bb5] Spek, A. L. (2020). *Acta Cryst.* E**76**, 1–11. 10.1107/S2056989019016244 PMC6944088 31921444

[bb6] Tannaci, J. F., Noji, M., McBee, J. L. & Tilley, T. D. (2008). *J. Org. Chem.* **73**, 7895–7900. 10.1021/jo8017268 18788782

